# Sistrunk Procedure on Malignant Thyroglossal Duct Cyst

**DOI:** 10.1155/2020/6985746

**Published:** 2020-01-16

**Authors:** Diani Kartini, Sonar S. Panigoro, Agnes S. Harahap

**Affiliations:** ^1^Oncology Division, Department of Surgery, Dr. Cipto Mangunkusumo General Hospital, Faculty of Medicine, Universitas Indonesia, Jakarta, Indonesia; ^2^Department of Anatomical Pathology, Dr. Cipto Mangunkusumo General Hospital, Faculty of Medicine, Universitas Indonesia, Jakarta, Indonesia

## Abstract

A thyroglossal duct cyst is a lesion that occurs as a result from failure of the thyroglossal duct to obliterate during fetal development. Malignant progression is a rare event that might occur in less than 1% of all cases. Because of its rarity, there are conflicting opinions regarding the management of the case. In the present study, a 46-year-old male presented with a painless neck mass that had increased in size over the last 6 months. There was no difficulty in swallowing and breathing, change in voice, significant weight loss, or any signs of hyperthyroidism. Laboratory workup showed that results were within normal limits. Thyroid gland ultrasonography and cervical contrast CT scan revealed a complex cystic mass that pointed towards a thyroglossal duct cyst. We performed Sistrunk procedure. Postoperative pathology examination revealed microscopic appearance of the thyroglossal duct cyst with a classic follicular variant of papillary thyroid carcinoma. Our latest follow-up showed no signs of tumor recurrence or any complications following surgery on locoregional status. As a fine needle aspiration biopsy cannot ensure a precise result in all of cases, it is essential to perform a solid physical examination and thorough supporting examination in deciding the precise management for the patient.

## 1. Introduction

A thyroglossal duct cyst (TGDC) is the most common form of congenital anomaly in thyroid development [1]. Approximately, 70% of midline neck masses in children and 7% of midline neck masses in adults are thyroglossal duct cysts [2]. Normally, during the third week of fetal development, the thyroid gland descends along the thyroglossal duct, a structure originating from the foramen caecum of the tongue, passing through the base of the tongue towards the lower front part of the neck, where it is usually found in adults. The thyroglossal duct physiologically disappears by the tenth week of gestation [3, 4]. In some cases, the thyroglossal duct may fail to obliterate and form a thyroglossal duct cyst [5]. Malignancy of the thyroglossal duct cyst rarely occurs, only in less than 1% of all cases, with papillary carcinoma presenting as the most common type [6]. This malignancy was first reported by Brentano in 1911 [7]. An estimate of up to 60% of cases are found in children less than 5 years of age. Nevertheless, up to one-third of cases may occur in patients aged 20 years and older [8, 9]. Dysphagia, dysphonia, weight loss, or rapid growth in size could be signs of malignancy [10]. However, this neoplasia is characterized by relatively nonaggressive property and rarely involves the thyroid gland and locoregional lymph node spread [7].

Sistrunk procedure is a common option chosen for the surgery of a thyroglossal duct cyst, especially for those who are categorized into low risk patients, which consists of excision of the thyroglossal duct cyst, the middle part of hyoid bone, and the surrounding tissue around the thyroglossal tract [11]. However, in high-risk patients, total thyroidectomy with radiotherapy iodine ablation must be considered [12, 13]. Due to the rarity of thyroglossal duct cyst carcinoma, there are conflicting opinions regarding the management approach for this case.

In this report, we present a rare case of papillary carcinoma arising in the thyroglossal duct cyst along with a review of the literature focusing on the management of malignancy in the thyroglossal duct cyst.

## 2. Case Illustration

A 46-year-old male came to our surgical oncology clinic presenting with a chief complaint of a lump in the anterior compartment of the neck, located in the front and slightly to the right, which the patient had noticed since a year ago and has been increasing in size in the last 6 months. The patient denied any complaints of difficulty in swallowing, difficulty in breathing, change in voice, significant weight loss, or any signs of hyperthyroidism. The patient reported to have a history of dyslipidemia, and during inpatient care, we discovered that the patient had hypertension. The patient also reported to have undergone sinus surgery twice in the last 20 years. There was no significant family history.

Physical examination on the patient showed a neck mass in the front area, in the midline slightly located to the right, mobile, with soft surface, painless, solid, with a well-defined border, and sized approximately 5 × 5 × 4 cm. During palpation, no lymph node enlargement was noted. Based on the findings above, the patient was given a working diagnosis of suspected benign right nontoxic goiter (*struma nodosa nontoxic*).

**Figure 1:**
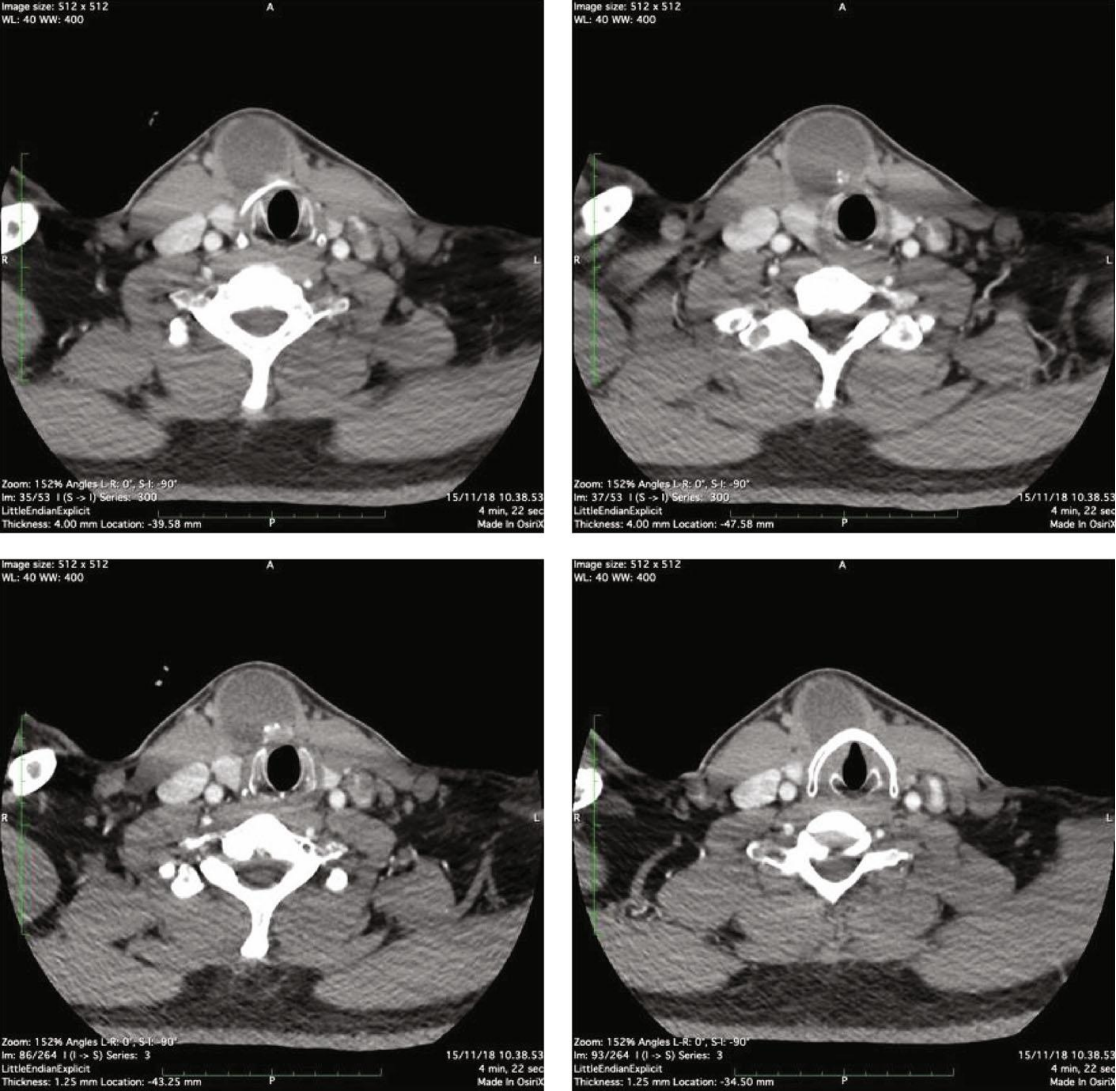
Cervical axial CT scan with contrast. Complex cystic mass in the right parasagittal anterior colli area, appears to bulge to the level of the larynx.

Thyroid gland ultrasonography was performed. The result showed that there was no abnormality in the thyroid gland; however, a cystic lesion in the anterior midline area was found, sized approximately 3.4 × 3.5 × 4.5 cm with debris sediment located inside the lesion which raised suspicions towards the thyroglossal duct cyst. To obtain a more accurate picture, a CT scan with contrast for the neck area was performed, which then showed a complex cystic mass in the front neck area located in the right parasagittal area, sized approximately 3.9 × 3.8 × 5.5 cm, attached to the right infrahyoid muscle, appearing to bulge into the larynx, with differential diagnosis of the thyroglossal duct cyst or epidermoid cyst (Figure 1). The thyroid gland appeared to be within normal limits. The scan also discovered multiple lymph node enlargement in the submental area and along the right jugular chain that measured largest in size 22 mm in the right area and 17 mm in the left. The thyroid function test was within normal limits.

**Figure 2:**
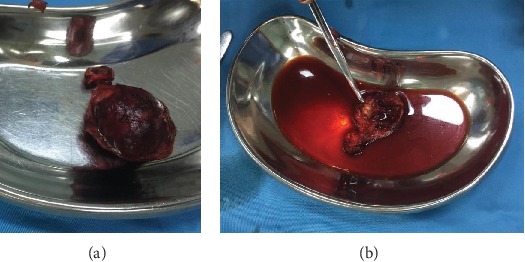
(a) Unopened excised specimen; (b) opened excised specimen.

**Figure 3:**
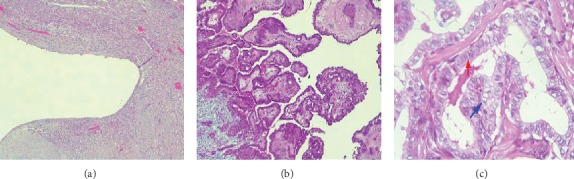
(a) Duct with a dense connective tissue wall lined with a layer of thorax epithelium that appears to be erosive (HE 40x); (b) tumor tissue with a papillary structure with a fibrovascular stalk (HE 100x); (c) tumor cells with an irregular nucleus with “ground glass nuclei” (blue arrow) and “nuclear grooves” (red arrow) (HE 400x).

The patient was then diagnosed with a thyroglossal duct cyst and planned to undergo Sistrunk procedure. The surgery went well, and the patient was discharged the day after. The excised specimen was then sent to pathology for histopathology examination. Macroscopic appearance of the specimen showed a cystic lesion that measured 4 × 3 × 3 cm with part of the specimen resembling white cauliflower and appeared to be fragile (Figure 2). Microscopic examination showed that the cystic lesion consisted of fibrous tissue lined by a single layer of epithelial cells that was erosive in most area. This area was the remnant of the thyroglossal duct cyst. Other areas showed that there were tumors in papillary structures with a fibrovascular stalk and some in a follicular pattern, lined by cells that were stratified, and showing ground glass nuclei, nuclear grooves, and eosinophilic cytoplasm (Figure 3). The histopathology conclusion was a thyroglossal duct cyst with a classic and follicular variant of papillary thyroid carcinoma.

On the 6-month follow-up after surgery, the patient reported no clinical complaints, and there were no locoregional complication nor clinically palpable lymph node enlargement; however, there was a lymph node enhancement discovered by cervical contrast CT scan, and the patient was planned for a lymph node biopsy; if the biopsy result showed to be malignant, selective neck dissection and total thyroidectomy would be the treatment of choice.

## 3. Discussion

The remnant structure of a thyroglossal duct can exist in the form of a cyst, tract, or duct or as ectopic thyroid tissue located inside the cyst or the duct [11]. The thyroglossal duct cyst is the most commonly found congenital anomaly of the neck in children aged less than 5 years old. An estimate of up to 60% of cases is found in children aged less than 5 years old, but almost one-third of cases can appear in patients aged 20 years and older [8, 9]. In our case, this patient belonged in the one-third with an age of 46 years old. Women were a little more likely to have this lesion compared to men, with a ratio of 3 : 2 [6]. The most common presenting chief complaint for the thyroglossal duct cyst is asymptomatic neck mass, sometimes accompanied by pain and dysphagia [14]. In our case, the only significant clinical finding was a painless mass in the neck, which increased in size during the last 6 months. Differential diagnosis of the thyroglossal duct cyst includes a branchial cleft cyst, lipoma, metastasis of thyroid carcinoma, dermoid cyst, sebaceous cyst, and lymph node enlargement [15].

CT scan, neck MRI, and ultrasonography hold an important role in preoperative diagnosis and management plan of the case [16], but imaging studies cannot ensure an accurate preoperative diagnosis, and a fine needle aspiration biopsy (FNAB) only gives accurate results in 66% of cases. According to a study by Yang et al., from 17 cases that are reported to have undergone FNAB, results showed 50% to be true positive and 47% to be false negative, mainly due to the hypocellularity of the first aspirate and dilution of the cyst [2]. Due to the cystic nature, FNAB is known to yield a low sensitivity and it has been reserved only for investigation of findings suspicious for malignancy such as the presence of calcifications or solid components on ultrasound [17]. Due to the low frequency of malignancy on the thyroglossal duct cyst, in the majority of cases, clinicians rarely consider diagnosis of malignancy, which caused FNAB to be rarely performed prior to surgery. However, the possibility of malignancy increases especially in the older population [18, 19]. Furthermore, FNAB does not rule out the presence of a malignancy especially if the clinical suspicion is high [20]. Generally, diagnosis for malignancy of the thyroglossal duct cyst can be made after the surgery with histopathology examination of the excised specimen [18]. In our case, we performed ultrasonography for the thyroid gland and CT scan for the neck area, and the result showed no signs of invasion of the capsule and the surrounding structure and also confirmed no abnormal findings regarding the thyroid gland. A conclusion was drawn from the physical and supporting examination in this case that it was a thyroglossal duct cyst with no signs of malignancy.

Progression towards malignancy rarely occurs in the thyroglossal duct cyst. The most frequent type of thyroglossal duct cysts is the papillary type (85%), followed by mixed papillary-follicular type (7%), squamous cell type (5%), and follicular type (1.7%), and also Hurthle cell and anaplastic type (0.9%) which has a worse prognosis [21, 22]. In our case, after the specimen was sent to pathology for histopathology examination, an interesting finding was brought to light that the type of the tumor did not resemble the most commonly found; rather, it resembled the second most common, which is the mixed papillary-follicular type.

Management for malignancy of the thyroglossal duct cyst is still widely debated, mainly when the thyroid gland remains in normal condition. Some authors support the theory that thyroglossal duct cyst malignancy originated from the ectopic thyroid tissue that is located inside the cyst, while some others think that the malignancy occurs from metastasis of the thyroid gland [6]. The same goes for the surgical management for the case; some authors think the Sistrunk procedure to be adequate if there is no abnormality in the thyroid gland.

Due to rarity of malignancy in the thyroglossal duct cyst, currently, there is no single universal guideline for the management of the case. Patients are classified into low risk and high risk according to the revised 2009 American Thyroid Association (ATA) guideline regarding differentiated thyroid cancer and National Comprehensive Cancer Network risk stratification system with proposed modifications adjusted to fit for malignancy in the thyroglossal duct cyst. Patients who are grouped into the low risk category are (1) patients aged between 15 and 45 years old, without prior radiation history; (2) tumor size less than 4 cm; and (3) no distant metastases or lymph node involvement. The majority of the patients fall into this category, and for those who present with no involvement of the thyroid gland or suspicious findings, Sistrunk procedure is considered adequate [9, 11–13, 23]. Patients who are grouped into the high risk category are (1) male patients; (2) patients aged 45 years old and above; (3) tumor size more than 4 cm; (4) presence of extracystic invasion; (5) presence of lymph node metastasis; (6) prior history of radiation, especially in the neck area; and (7) presence of cold nodules in thyroid gland imaging [19]. Definitive management on thyroglossal duct cyst malignancy remains controversial. Some experts considered Sistrunk procedure to be adequate and curative for majority of cases, while few others considered that total thyroidectomy must be carried out on thyroglossal duct cyst malignancy due to the high incidence of papillary carcinoma or mixed type carcinoma on the thyroid gland [24, 25].

There are four approaches regarding surgical management for thyroglossal duct cyst malignancy, which are (1) Sistrunk procedure alone [8], (2) Sistrunk procedure with thyroid lobectomy or pyramidal lobe resection [26], (3) Sistrunk procedure with total or near total thyroidectomy in all patients [9, 27], and (4) Sistrunk procedure with selective thyroidectomy for high-risk patients [28, 29]. Consideration to adding thyroid resection in all patients is based on 3 aspects: (1) presence of thyroid malignancy, (2) use of radioactive iodine as adjuvant therapy, and (3) role of thyroglobulin as a follow-up marker [9]. Sistrunk procedure was introduced in 1920 by Sistrunk which consists of resection of the cyst, its tract, the middle part of hyoid bone, and the structure that composes the base of the tongue. By using this procedure, the recurrence rate could be decreased significantly compared to simple excision: from 40% (simple excision) to 1-5% (Sistrunk procedure) [30–32]. Based on the study by Balallaa et al., total thyroidectomy is indicated without considering the presence of thyroid gland involvement clinically or radiologically based on the premise that this procedure could assist staging and detect metastasis, and the risk of recurrent laryngeal nerve injury or parathyroid gland injury is considerably low especially on the hands of an experienced operator [11]. In this case, we only performed Sistrunk procedure, considering that there was no pathological diagnosis, no thyroid nodule on physical and supporting examination, and no cervical lymph node enlargement noted.

## 4. Conclusions

Malignancy in a thyroglossal duct cyst is a rare event. Physical and supporting examinations are both crucial in deciding how the surgical procedure must be carried out. In our case, we would continue to observe the locoregional status by both physical and supporting examinations.
